# Questionnaire study of application about sentinel lymph node biopsy surgery in locally advanced breast cancer patients who received neoadjuvant chemotherapy

**DOI:** 10.3389/fonc.2023.1235938

**Published:** 2023-10-02

**Authors:** Eun-Gyeong Lee, Minjung Lee, So-Youn Jung, Jai Hong Han, Seok-Ki Kim, Seeyoun Lee

**Affiliations:** ^1^ Department of Surgery, Research Institute and Hospital, National Cancer Center, Goyang, Republic of Korea; ^2^ Department of Nuclear Medicine, Research Institute and Hospital, National Cancer Center, Goyang, Republic of Korea

**Keywords:** minimally invasive surgery, neoadjuvant chemotherapy, sentinel lymph node biopsy, breast cancer, questionnaire

## Abstract

**Background:**

Nodal staging from sentinel lymph node (SLN) biopsy has become the standard procedure for early-stage breast cancer patients. SLN biopsy implementation after chemotherapy has previously been evaluated. This questionnaire study aimed to investigate the current trend of SLN biopsy after neoadjuvant chemotherapy (NAC) for locally advanced breast cancer.

**Methods and materials:**

We conducted a web-based survey among breast surgeons who are members of the Korean Breast Cancer Society. The survey comprised 14 questions about axillary surgery after NAC.

**Results:**

Of 135 respondents, 48.1% used a combined method of dye and radioactive isotope (RI). In the absence of SLN metastasis, 67.7% would perform only SLN biopsy, while 3% would perform ALN dissection. In case of SLN metastasis, the proportions of surgeons who would proceed with ALN dissection were 60.2% and 67.2% for less than two and more than three positive SLNs, respectively.

**Conclusion:**

The present study confirmed the increasing tendency to adopt SLN biopsy for axillary staging in patients who achieved complete response with initial nodal metastasis. It could be expected that the mapping methods for patients receiving NAC have become diverse, including RI, vital dye, and indocyanine green fluorescence. The implementation of SLN biopsy after NAC will grow in the coming years due to an increasing demand of minimally invasive surgery.

## 1 Introduction

Axillary lymph node (ALN) status represents an important prognostic factor in breast cancer patients without distant metastasis. The accurate assessment of nodal status is paramount to tumor staging and optimal treatment decision. Sentinel lymph node (SLN) biopsy has become the standard surgical procedure for axillary staging with fewer complications compared to ALN dissection (ALND). However, most studies reported that SLN surgery was performed for patients in early clinical stages (1, 2). Advanced breast cancer patients, particularly those with lymph node metastasis, undergo neoadjuvant chemotherapy (NAC) to downstage the tumor, which may allow SLN biopsy if the initial nodal disease becomes node-negative after NAC (3–6).

In several prospective trials, a relatively lower detection rate and a higher false negative rate (FNR) were observed in SLN biopsy after NAC as compared to upfront surgery (7–10). Therefore, the consensus of SLN biopsy after NAC was made with more than two mapping procedures and more than three harvested SLNs. A previous longitudinal analysis showed an increasing tendency to incorporate SLN biopsy in node-negative patients after NAC with initial node metastasis (11). Although adherence to traditional treatment modality was not changed, subsequent SLN biopsy implementation for patients with complete nodal response after NAC could be expected.

This study aimed to investigate the application of SLN biopsy after NAC for locally advanced breast cancer patients in the current clinical setting by conducting a web-based survey among breast surgeons who are members of the Korean Breast Cancer Society (KBCS).

## 2 Materials and methods

### 2.1 Study design and participants

We conducted a survey of KBCS members from December 2020 to November 2021. The questionnaire could be accessed with an online link for the Google survey form. The participants were breast cancer surgeons who practice in Korea. We collected information on the clinical experience of the respondents and the size of hospitals (regarding the number of beds and the number of breast surgeons in their hospitals). The questionnaire comprised 14 questions about axillary surgery. This study was approved by the Institutional Review Board of the National Cancer Center (approval no. NCC 2020-0338).

### 2.2 Questionnaire statements

The participants were provided with questions regarding the following topics (1): surgical procedures for patients with NAC response in preoperative imaging examination (2), mapping methods for axillary staging in patients with axillary metastatic breast cancer [mapping using indocyanine green fluorescence (ICG-F) with a near-infrared (NIR) camera and its applicability were also surveyed] (3), surgical procedure for unidentified SLNs (4), surgical procedures when SLN metastasis from SLN biopsy was absent (5), surgical procedures for metastasis to less than two SLNs as a result of intraoperative biopsy (6), surgical procedures for metastasis to less than two SLNs as a result of a final pathologic report, and (7) surgical procedures for metastasis to more than three SLNs as a result of final pathology.

### 2.3 Statistical analysis

This questionnaire study was descriptive by nature. The sample size was calculated to be 580, considering the maximum sample size of 1,160 (the number of surgeons who are KBCS members) and a response rate of 50%. The datasets generated or analyzed during this study are available from the corresponding author on reasonable request. The responses to the questionnaire were presented by domain, along with the sample size (*N*) and percentage breakdown based on the general characteristics of the survey participants. For categorical variables, percentages were calculated using tests such as the chi-square test or Fisher’s exact test.

## 3 Results

**Figure 1:**
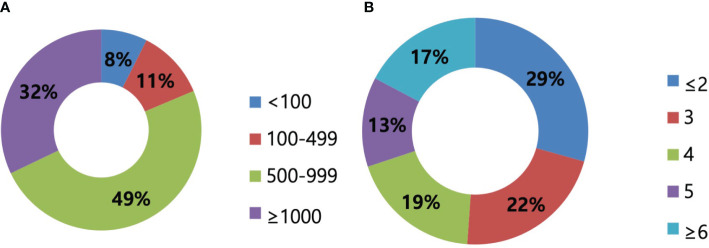
Size of hospitals. **(A)** The size of hospitals regarding the number of beds presented. In total, 49% of respondents worked in hospitals with 500–999 beds, followed by 32% of more than 1,000 beds. **(B)** The size of hospitals regarding the number of surgeons presented. The majority of respondents worked in hospitals with less than two surgeons, and the median number was 3.

**Figure 2:**
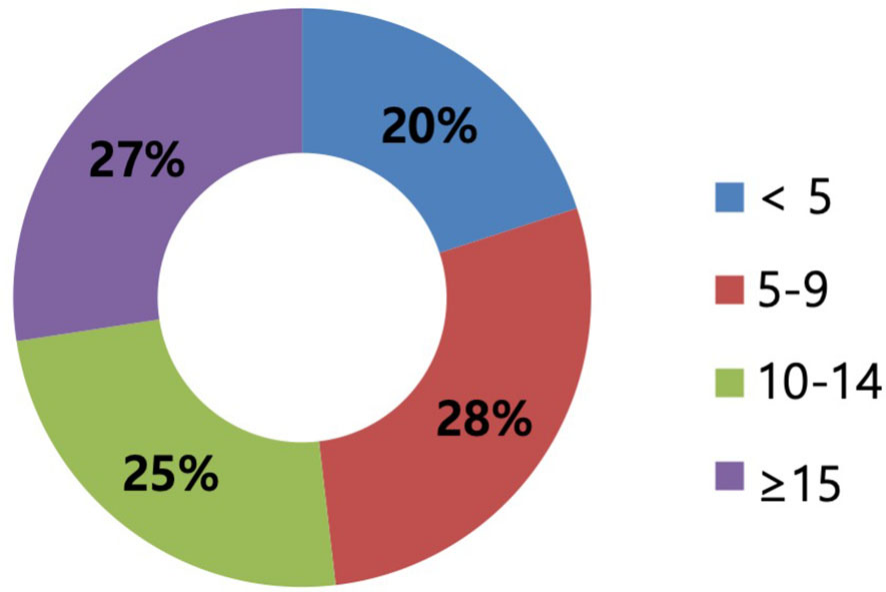
The experience as surgeons of respondents (years). The years of experience are categorized by a 5-year interval scale corresponding to less than 5, 5–9 years, 10–14 years, and more than 15 years. The experience as surgeons varied and was evenly distributed.

A total of 135 surgeons out of 1,160 KBCS members (11.6%) provided survey responses. Most participants were from hospitals with <1,000 beds, and 32.1% were from hospitals with >1,000 beds (**Figures 1A, B**). While the surgical experience of the respondents varied, it was evenly distributed, with 51.8% of the respondents having >10 years of experience as breast surgeons (**Figure 2**).

**Figure 3:**
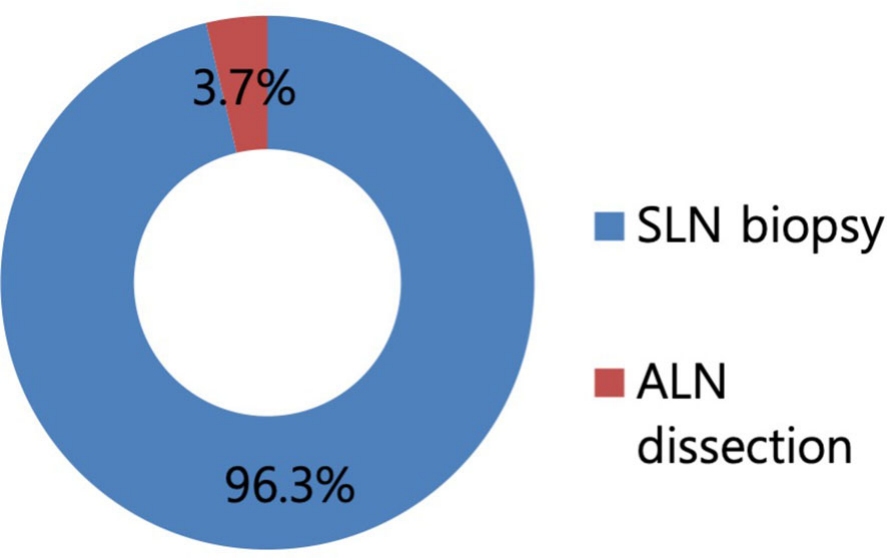
Proportion of surgical procedures in case with the response to neoadjuvant chemotherapy (NAC) in a preoperative imaging study. In total, 96.3% of respondents answered to perform sentinel lymph node biopsy first in case with the response to NAC in a preoperative imaging study.

**Figure 4:**
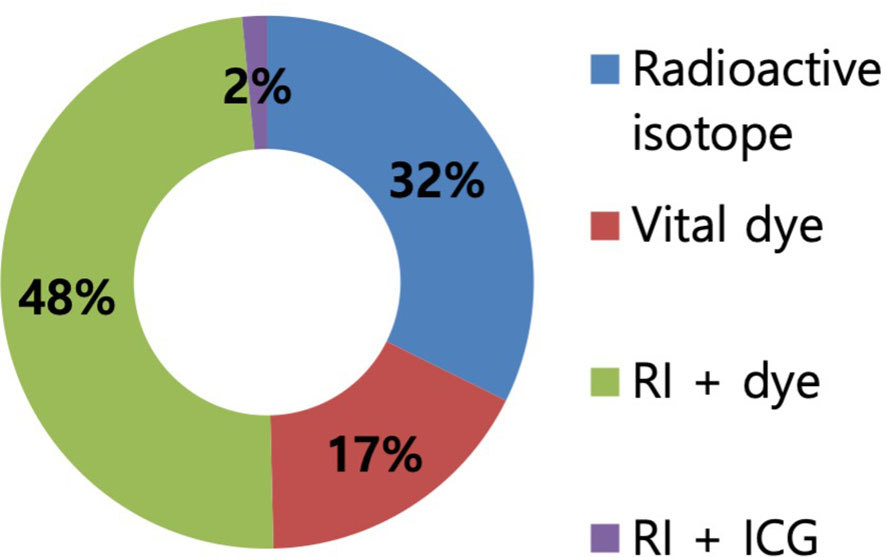
Mapping methods for the evaluation of axillary stage in patients with axillary metastatic breast cancer after neoadjuvant chemotherapy. In total, 48% of respondents answered to use the dual method of radioactive isotope (RI) and dye to axillary mapping, followed by 32% of RI alone, 17% of dye alone, and 2% of RI and indocyanine green.

**Figure 5:**
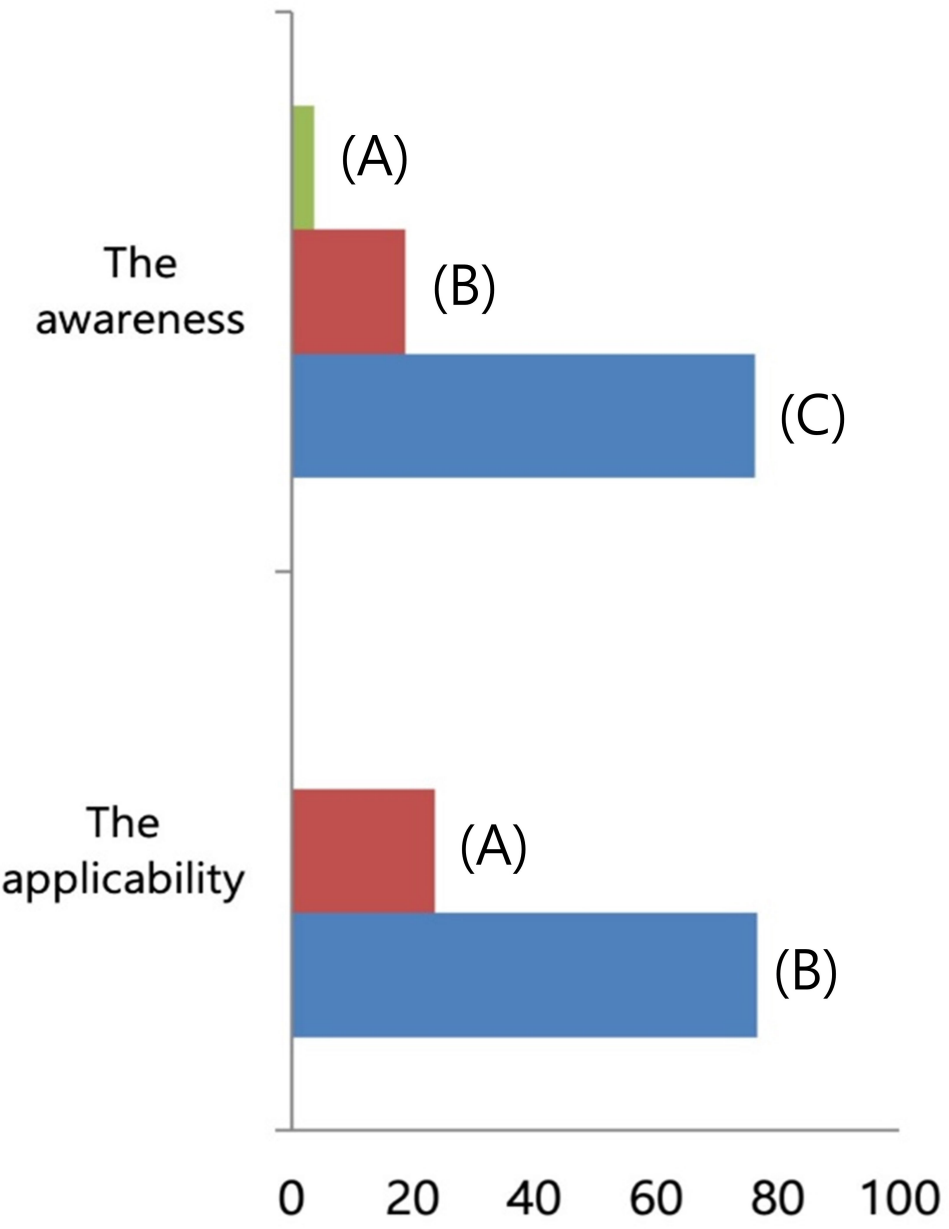
The familiarity of indocyanine green (ICG) with a near-infrared (NIR) camera as a mapping method. (Above) To the question about the familiarity of ICG with NIR camera, **(A)** 3.7% of respondents answered to have not known the method, **(B)** 18.7% with experiences to use it, and **(C)** 76.1% with knowledge. (Below) To the question about the applicability of ICG with NIR camera, **(A)** 23.5% of respondents answered to avoid it and **(B)** 76.5% had interest to apply it as a mapping method.

**Figure 6:**
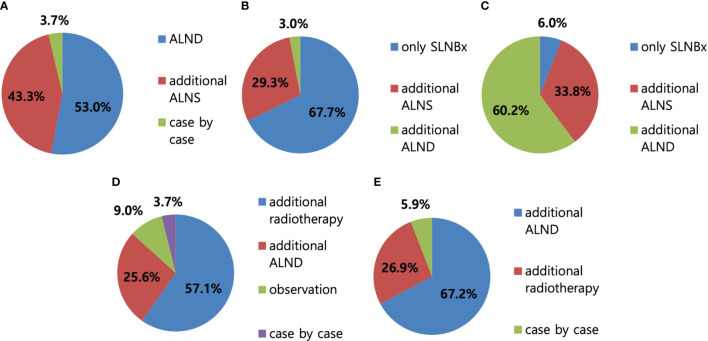
Axillary surgery after neoadjuvant chemotherapy (NAC). **(A)** In case of unidentified sentinel lymph node (SLN), 53% of respondents answered to perform axillary lymph node dissection (ALND), followed by 43.3% of axillary lymph node (ALN) sampling. **(B)** In case of the absence of SLN metastasis with SLN biopsy, 67.7% of respondents answered to perform SLN biopsy only, followed by 29.3% and 3% of ALN sampling and ALND, respectively. **(C)** In case of less than two SLN metastasis with SLN biopsy, 60.2% of respondents answered to perform ALND first, followed by 33.8% and 6% of ALN sampling and SLN biopsy, respectively. **(D)** In case of less than two SLN metastasis on the final pathologic report, 57.1% of respondents answered to proceed with radiotherapy, followed by 25.6% and 9% of ALND and observation without further treatment, respectively. **(E)** In case of more than three SLN metastasis on the final pathologic report, 67.2% of respondents answered to proceed with additional ALND surgery, followed by 26.9% of radiotherapy.

Regarding the surgical procedures for patients with NAC response in preoperative imaging examination, 96.3% of the respondents would perform SLN biopsy first (**Figure 3**). Regarding the mapping methods for axillary staging in patients with axillary metastatic breast cancer, 48.1% of the respondents used the combined method of dye and RI, followed by 32% who used RI alone and 17.3% who used dye alone. Two surgeons answered that they would use RI with the addition of vital dye if the former was insufficient to detect SLNs (**Figure 4**). Regarding the mapping method of ICG-F with a NIR camera, 76.1% of the respondents were aware of this mapping method, 18.7% had performed this method, 76.5% of respondents would elect this method, and 23.5% of surgeons would avoid it. The following question showed that the cost of purchasing and setting up an NIR camera was a roadblock to applying this method in a cost-effective manner (**Figure 5**). Regarding unidentified SLNs after NAC, 53% of the respondents would perform ALN dissection, and 43.3% would dissect suspicious lymph nodes (ALN sampling, ALNS). Some surgeons would make their decisions in consideration of the initial stage, NAC response, and the presence of initial fine needle aspiration biopsy. Regarding surgical procedures when SLN metastasis from SLN biopsy was absent, 67.7% of the respondents would end the surgery, 29.3% would proceed with additional lymph node sampling, and 3% would perform ALN dissection. Regarding surgical procedures for metastasis to less than two SLNs as a result of intraoperative biopsy, 60.2% of the respondents would perform ALN dissection, 33.8% would perform ALNS, and 6% would end the surgery without further dissection. Regarding surgical procedures for metastasis to less than two SLNs as a result of final pathology, 57.1% of the respondents would add adjuvant radiotherapy, while 25.6% would proceed with ALN dissection. Some surgeons answered that they would consider the number of harvested lymph nodes or the ratio. Regarding surgical procedures for metastasis to more than three SLNs as a result of final pathology, 67.2% of the respondents would perform additional ALN dissection, while 26.9% would add axillary radiotherapy (**Figures 6A–E**).

## 4 Discussion

SLN biopsy has been adopted for axillary staging in patients with node-negative early breast cancer. Since NAC was widely accepted as a method to downgrade the tumor in order to reduce the surgical burden, a question has been raised on whether SLN biopsy could replace conventional ALN dissection in patients with initial node metastasis. Several studies were conducted to determine the accuracy of SLN biopsy after NAC (3, 7–9, 12, 13). In the SENTINA prospective, a multicenter cohort study, quadripartite participants according to clinical axillary nodal status were evaluated to determine the accuracy of SLN biopsy before and after chemotherapy. Among 715 (41%) of 1,737 patients with initially suspicious nodes who underwent NAC, 592 patients (83%) converted to node-negative. SLN detection rate using lymphoscintigraphy was 80.4% in this patient group, which was lower than the rate of 99.2% in the group of patients with SLN biopsy before NAC. For the combined mapping technique with the additional use of blue dye, the detection rate reportedly increased to 87.8%, and a higher number of SLN was detected, which significantly lowered the FNR (16.0% with radio-colloid alone vs. 8.6% with radio-colloid and blue dye). Patients who had removal of one SLN had an FNR of 24.3%, while those who had removal of more than three SLNs had an FNR of <10% (10). In the protocol B32 study of the National Surgical Adjuvant Breast and Bowel Project (NSABP), FNR increased to 17.7% with the detection of one lymph node (LN), while FNR was 10.0% with the removal of more than two SLNs (14). The American College of Surgeons Oncology Group (ACOSOG) Z1071 trial also showed FNR of 12.6% after NAC with ≥2 SLNs, which significantly decreased with the combined use of blue dye and radiolabeled colloid (*P* = 0.05, 10.8% with combined use vs. 20.3% with single agent) (7). These findings led us to focus on the improvement of the detection rate and the number of SLNs. In 2018, many medical centers began to adopt the practice that routine ALND should not be the standard treatment for SLN-positive patients, except in cases where radiotherapy is not an option (15).

To date, the optimal material for SLN biopsy has been studied to improve the identification rate of SLNs (16). Blue dye and RI were widely used as single agents or in combination. Blue dye reportedly had higher FNR than that of RI or the combination method. On the other hand, the RI method required an injection technique and lymphoscintigraphy. ICG was introduced as an alternative method with a reported detection rate of 98% and a sensitivity rate of 92%. However, ICG could spread to adjacent tissues to yield an increase in the number of SLNs. Recently, ICG-F showed a significantly better identification rate of SLNs than blue dye, similar to that of RI. In our phase II, prospective case–control study (17), the feasibility of ICG-F following detection using NIR light source combined with RI as a dual mapping method was demonstrated. In this study, the identification rate of ICG-F and RI was higher than that of RI alone (98.3% vs. 94.7%). Another study of ours has been conducted to investigate the differences between several dual methods, with the combination of ICG-F, RI, and blue dye, for SLN mapping, for which we anticipated promising results. Regarding the familiarity of the respondents with ICG-F with the NIR camera, 18.7% had used this method, 76.1% had heard of this method without using it, and 76.5% would consider the application of ICG-F with the NIR camera. As such, we can expect that this method for axillary staging surgery could be adopted more.

In this study, we asked the KBCS members about the methods and procedures for axillary staging after NAC in patients with locally advanced breast cancer by a web-based questionnaire. For patients with NAC response in preoperative imaging study, 96.3% of the surgeons would perform SLN biopsy first. Almost half of the surgeons chose dual mapping with RI and dye in this setting. In the absence of SLN metastasis, 67.7% of the surgeons would not proceed with further dissection, while 29.3% would conduct ALNS and 3% would perform ALN dissection from the beginning. Most surgeons would elect additional ALN dissection in case of SLN metastasis, or axillary radiotherapy was favored if metastasis was identified as a result of final pathology. Lee et al. documented a change in surgeons’ behaviors with increasing adoption of SLN biopsy in patients with node-negative conversion after NAC between 2013 and 2017. The proportion of surgeons in preference of SLN biopsy increased from 53.5% in 2013 to 92.1% in 2017. In 2013, 53.5% of the surgeons would prefer additional ALN dissection, and this figure decreased to 36.8% in 2017 in the absence of SLN metastasis. A similar trend to apply minimal axillary surgery in the current clinical setting was observed in this study. Only 3% of the respondents would perform ALN dissection first in the absence of SLN metastasis. It might be affected from previous studies with the feasibility of SLN biopsy by using combined mapping procedures. Another possible reason for the decreasing trend of ALN dissection might be the influence of NAC, which normalizes axillary LNs in preoperative imaging examination. Without residual suspicious LNs, surgeons would not prefer to perform ALN dissection but would choose additional ALNS in consideration of LN ratio. In the aspect of surgeon’s experience, 5.8% of the respondents who had >10 years of clinical experience as breast surgeons would perform ALN dissection in the absence of SLN metastasis with SLN biopsy, while 1.6% of those with 5–10 years of experience would conduct ALN dissection.

Our study had some limitations, given the survey nature of the study. First, to simplify the survey, we did not consider the influence of demographic and clinical parameters, such as age, tumor histology, and risk factors. Second, we could not evaluate in detail the determinants of the decision-making process of the participants. Third, the number of participants was small, which could be explained by the fact that, among the KBCS members, the total number of surgeons in large hospitals who perform NAC was relatively lower than our estimate.

In conclusion, our findings confirmed the increasing tendency to adopt SLN biopsy as axillary staging in patients who achieved complete response with initial nodal metastasis. It could be expected that the mapping methods for patients who underwent NAC have become diverse, including RI, vital dye, and ICG-F. The implementation of SLN biopsy after NAC will likely grow in the coming years due to the increasing demand of minimally invasive surgery.

## Data availability statement

The datasets presented in this study can be found in online repositories. The names of the repository/repositories and accession number(s) can be found below: Not applicable.

## Ethics statement

The Institutional Review Board of our institute approved the study protocol (approval number: NCC 2020-0338). This study was conducted in accordance with the guidelines of the Declaration of Helsinki. Written informed consent was waived due to the nature of the questionnaire study.

## Author contributions

Research design: SL, S-YJ, JH, S-KK, and E-GL. Writing or critical revision of the paper: E-GL, ML, and SL. Data analysis: ML, E-GL, and SL. All authors contributed to the article and approved the submitted version.
